# Venous thromboembolism in women: new challenges for an old disease

**DOI:** 10.1590/1677-5449.190148

**Published:** 2020-07-06

**Authors:** André Luiz Malavasi Longo de Oliveira, Adilson Ferraz Paschôa, Marcos Arêas Marques

**Affiliations:** 1 Centro de Referência da Saúde da Mulher do Estado de São Paulo, Obstetrícia e Ginecologia, São Paulo, SP, Brasil.; 2 Hospital da Beneficência Portuguesa de São Paulo, Cirurgia Vascular, São Paulo, SP, Brasil.; 3 Universidade do Estado do Rio de Janeiro – UERJ, Hospital Universitário Pedro Ernesto, Unidade Docente Assistencial de Angiologia, Rio de Janeiro, RJ, Brasil.; 4 Universidade Federal do Estado do Rio de Janeiro – UNIRIO, Hospital Universitário Gafrée e Guinle, Serviço de Cirurgia Vascular, Rio de Janeiro, RJ, Brasil.

**Keywords:** thrombosis, contraceptive agents, pregnancy, hormone replacement therapy, women, pulmonary embolism

## Abstract

In countries that have controlled classic causes of maternal death, such as eclampsia and hemorrhage, venous thromboembolism (VTE) has become the major concern. Prevention of VTE during pregnancy and postpartum by applying guidelines and implementing pharmacoprophylaxis is still the best strategy to reduce occurrence of this complication. Hormonal contraceptives and hormone replacement therapy also increase the risk of VTE, but women cannot be deprived of their benefits, which increase their freedom at childbearing age and reduce their symptoms at menopause. Both indiscriminate use and unmotivated prohibition are inappropriate. Contraceptive and hormone replacement methods should be chosen with care, evaluating the patients’ contraindications, eligibility criteria, and autonomy. This article presents a nonsystematic review of recent literature with the aim of evaluating and summarizing the associations between VTE and clinical situations peculiar to women.

## VENOUS THROMBOEMBOLISM IN PREGNANCY

Modern obstetrics is faced by a bleeding-thromboembolism duality dilemma. If, in the not too distant past the greatest dread among those who cared for women giving birth was accidental hemorrhage, nowadays, this fear is compounded by another complication: venous thromboembolism (VTE).^1^^,^^2^

Pregnant women have all three etiopathogenic components of Virchow’s triad: a) venous stasis, caused by compression of the vena cava and left common iliac vein by the gravid uterus and by reduced venous tone because of the myorelaxant action of progesterone; b) hypercoagulability, secondary to induction of hepatic synthesis of coagulation factors VII, VIII, and X by placental estriol, increased levels of fibrinogen and plasminogen activator inhibitor types I and II, and reduced synthesis of protein S; c) endothelial injury, which occurs during nidation, endovascular remodeling of uterine spiral arteries, and expulsion of the placenta.^3^

During pregnancy, the risk of VTE increases by between five and ten times, and can be 35 times higher during the puerperium, when compared with the rate among women of the same age who are not pregnant.^4^ After this period, the frequency reduces rapidly; but there is a residual risk for up to 12 weeks after delivery.^5^

Deep venous thrombosis (DVT) in the lower limbs is responsible for 75 to 80% of VTE episodes in pregnancy. Approximately two-thirds of DVTs occur during the prenatal period, equally distributed across the three trimesters of gestation. However, 43 to 60% of pulmonary embolism (PE) episodes occur during the first 6 weeks of the puerperium.^6^ When compared with women who are not pregnant, among pregnant women DVTs in the left lower limb (90% vs. 55%) and the iliofemoral segment (72% vs. 9%) are even more predominant. This is because of the accentuated compression of the left common iliac vein against the fifth lumbar vertebra by the right common iliac artery, caused by the gravid uterus.^3^

The prevalence of VTE is in the range of 0.5 to 2.2 cases per 1,000 deliveries, depending on the study population,^7^ and the absolute incidence of VTE during pregnancy and the puerperium was 107 per 100,000 woman-years in the United Kingdom (UK)^8^ and 175 per 100,000 woman-years in Denmark and in Canada.^9^ There are no official data on maternal VTE mortality for Brazil.^6^

Although PE remains the number one cause of direct maternal death in the UK, there was a significant fall in the rate linked with vaginal deliveries, from 1.56 per 100,000 deliveries in 2003-2005 to 0.70 per 100,000 deliveries in 2006-2008, which can be attributed to application of the first version (2004) of the Royal College of Obstetricians and Gynaecologists’ (RCOG) guideline on reducing the risk of VTE in pregnancy and the puerperium.^10^ Prevention of VTE in pregnancy, by means of application of guidelines and the consequent implementation of mechanical and/or pharmacological prophylaxis is the best strategy for reducing the rate of these events.^6^

## RISK FACTORS FOR VTE DURING PREGNANCY

### Overweight and obesity

Obesity (body mass index [BMI] > 30 kg/m^2^) is an important risk factor for VTE in pregnancy and the risk grows as BMI increases,^11^ reaching as much as 14.9 times higher than the risk for women who are not obese.^12^ Sixty percent of the pregnant women who died from PE in the UK from 2003 to 2008 were obese.^10^ While maternal overweight (BMI from 25 to 29.9 kg/m^2^) is common, it is considered a less important risk factor for pregnancy-related VTE.^12^

### Age

Data extracted from studies with case-control designs suggest that women over the age of 35 years are at double the risk.^13^ In a study conducted in the UK, using a broad cohort of women who were not pregnant, those aged 35 to 44 years had a 50% higher risk of VTE when compared with those aged 25 to 34 years. The prenatal VTE rate did not increase with age, but women aged 35 to 44 years exhibited a 70% greater risk in the puerperal period when compared with those aged 25 to 34 years (an increase in absolute risk of 1.6 per 1,000 person-years).^8^ In general, age of 35 years or more is considered an antenatal and postnatal risk factor.^14^

### Thrombophilias

Although there is an association between hereditary thrombophilias and VTE, systematic work-up for thrombophilias should not be conducted for all women, but restricted to patients with prior episodes or family history of thrombophilias with greater potential for complications, such as antithrombin deficiency and homozygosis of factor V Leiden and the 20210 prothrombin mutation, which may need VTE prophylaxis. Depending on the circumstances, this may be prescribed from the start of pregnancy up to puerperium, from the 28th week onwards, or just during puerperium. It is important to point out that clinical history is of greater relevance for this decision than the results of laboratory tests. In turn, the association between thrombophilias of genetic causes and uteroplacental thrombosis causing miscarriage, fetal growth restriction, or placental abruption, remains a controversial subject.^15^^-^17

### Long-distance travel and immobility

There are limited data on immobility and long-distance travel in pregnant women, so it is necessary to extrapolate from studies of populations women pregnant who are not.^18^ Guidelines from the UK’s National Institute of Health and Care Excellence (NICE)^19^ and the recommendations of the RCOG on flying during gestation state that flights with duration exceeding 4 hours increase the risk of VTE. A Norwegian case-control study identified an increased risk of VTE in pregnant women with BMI > 25 kg/m^2^ and prepartum immobilization (defined as confinement to bed for a period greater than or equal to 1 week before birth or before diagnosis of VTE), showing a multiplication effect on prenatal and postnatal risk of VTE.^11^

Hospital admission during pregnancy is associated with an 18-times increase in the risk of VTE compared with the baseline risk outside of hospital and the risk remains elevated after birth, for the next 28 days. When admitted to hospital, the risk is higher in the third trimester of pregnancy and in women over the age of 35 years.^20^

### Others

Certain comorbidities have been associated with increased risk of VTE during pregnancy. These include inflammatory intestinal disease, urinary tract infection, systemic lupus erythematosus, heart diseases, pregnancy-induced systemic arterial hypertension or pre-eclampsia, and non-obstetric antenatal surgery.^21^ In an analysis of data from 1,475,301 discharges from Scottish hospital maternity units, Kane et al. found that risk factors associated with VTE included three or more previous gestations, obstetric hemorrhage, and pre-eclampsia.^21^ Hyperemesis increases prenatal and postnatal risk of VTE 4.4 times.

This information has important implications, since many thromboembolic events are fatal and occur during the first trimester, often before the first prenatal consultation, when antenatal prophylaxis may be initiated.^22^

### Factors related to anticoagulant treatment during gestation and puerperium

Administration of warfarin during pregnancy can induce placental abruption, embryopathy, abnormalities of the central nervous system (CNS), and fetal bleeding. Warfarin embryopathy is characterized by nasal hypoplasia and/or stippling of the epiphyses and is associated with exposure to warfarin between the sixth and twelfth weeks of gestation.^23^ Central nervous system abnormalities associated with use of warfarin include dysplasia of the dorsal midline with agenesis of the corpus callosum, atrophy of the cerebellar midline, dysplasia of the ventral midline with optical atrophy and amaurosis, and hemorrhage, and can occur after exposure to warfarin at any phase of the gestation. Warfarin is safe when breastfeeding.^24^

Two approaches can be taken to reduce the risk of warfarin use in women who need anticoagulation and intend to become pregnant. The first is to maintain warfarin and conduct pregnancy tests frequently. As soon as pregnancy is detected, before the sixth week, low molecular weight heparin (LMWH) should be introduced to replace warfarin. The other approach is to withdraw warfarin and start LMWH as soon as the decision to become pregnant is taken.^24^^-^28

Although existing direct oral anticoagulants (apixaban, dabigatran, edoxaban, and rivaroxaban) have enabled major advances in prophylaxis and treatment of VTE in many clinical situations, they are contraindicated in pregnancy, because they cross the placental barrier, and in breastfeeding, because they pass into breast milk.^6^^,^^23^^-^25

### Caesarean

Although the risk of VTE associated with caesarean in isolation is low, the rate of VTE occurrence becomes significant when there is a relationship with other risk factors, and so thromboprophylaxis should be prescribed.^29^ Delivery of anticoagulated pregnant woman should be scheduled for 37 to 40 weeks. Low molecular weight heparin should be withdrawn 12 hours before delivery if given at prophylactic dosages, or 24 hours before if administered at intermediate or full dosages, enabling safe administration of spinal or epidural anesthesia. Pregnant women on LMWH should be instructed not to administer the dose if they experience contractions or release of liquids.^23^

The choice of delivery route is obstetric and there is no contraindication against artificial cervical ripening or induction of labor. The patient should continue wearing antiembolism stockings throughout the procedure, regardless of whether delivery is vaginal or caesarean.

### Risk stratification

Stratification of VTE risk in pregnancy is based on assessment of each patient and should be performed for all women who intend to become pregnant or as soon as they become pregnant. It is recommended that assessments are repeated throughout the prenatal period, since new risk factors could emerge. The patient’s preferences and views should be taken into account when choosing thromboprophylaxis, even though the treatment options are restricted in this situation.^26^

The recommendations suggested are based on individual variations between patients and are intended to inform, rather than substitute, the physician’s clinical judgment, which should determine the appropriate management for each case in the final analysis. The most relevant guidelines on the subject of diagnosis, prophylaxis, and treatment of VTE in pregnancy are those published by the American College of Obstetricians and Gynecologists (ACOG),^30^ the Society of Obstetricians and Gynaecologists of Canada (SOGC),^31^ the RCOG,^27^ and the American College of Chest Physicians (ACCP).^28^^,^^29^

### Summary of guidelines

As mentioned above, the objective of the guidelines is to set out the most appropriate conduct on the basis of publications available in the literature. Notwithstanding, atypical situations are common and demand different treatment. The major guidelines are summarized below.

**ACOG:** intermittent pneumatic compression (IPC) before caesarean if the patient is not on thromboprophylaxis.^30^

**SOGC:** drug-based thromboprophylaxis is recommended postpartum in the following situations: prior VTE, high risk thrombophilia (antiphospholipid antibody syndrome, antithrombin deficiency, homozygosis of factor V Leiden or the G20210A mutation of the prothrombin gene, or combined thrombophilias), confinement to bed for 7 or days or more before delivery, bleeding exceeding 1 L during peripartum or postpartum, transfusion of blood products, postpartum surgery, and infection during peripartum or postpartum.^31^

Pharmacological thromboprophylaxis should be considered in the presence of two or more of the following: BMI ≥ 30 kg/m^2^ at first prenatal consultation, smoking > 10 cigarettes/day, preeclampsia, fetal growth restriction, placenta previa, emergency caesarean, bleeding exceeding 1 L during peripartum or postpartum or transfusion of blood products, low-risk thrombophilia (deficiency of protein C or S, heterozygosis of factor V Leiden or the 20210A mutation of the prothrombin gene), maternal cardiac disease, systemic lupus erythematosus, sickle-cell anemia, intestinal inflammatory disease, varicose veins of the lower limbs, gestational diabetes, premature delivery, stillbirth; or three or more of the following risk factors: age > 35 years, parity ≥ 2, any type of assisted reproduction technique, multiple gestation, placental abruption, premature rupture of membranes, elective caesarean, or maternal cancer.

Women with persistent risk factors should be given pharmacological thromboprophylaxis for a minimum of 6 weeks postpartum, while those with transitory factors during prepartum and peripartum should be given pharmacological thromboprophylaxis up to hospital discharge, or 2 weeks postpartum.^25^

**RCOG:** emergency caesarean, for 10 days postpartum with prophylactic doses of LMWH; for all other patients delivered by caesarean, consider 10 days of LMWH at prophylactic doses if other risk factors are present.^27^

**ACCP:** in the absence of additional risk factors, early mobilization; in the case of one major risk factor or two or more minor ones (or one minor factor if emergency caesarean has been performed), prophylaxis with LMWH after delivery is suggested while the patient is still in hospital (in the presence of contraindications to anticoagulation, use mechanical prophylaxis with antiembolism stockings or IPC); in extremely high-risk cases, with additional risk factors that remain during puerperium, combine LMWH with antiembolism stockings and/or IPC; selected high risk patients with additional risk factors that remain during puerperium should be given up to 6 weeks extended pharmacological thromboprophylaxis after hospital discharge.^28^^,^^29^

### Venous thromboembolism and contraception

In Brazil, one in every five women use (OC).^32^ Oral contraceptives offer benefits that go beyond contraception, such as reduction of menstrual bleeding, dysmenorrhea, treatment of premenstrual syndrome, menstrual migraine, acne, and hirsutism. The long-term benefits include reduced rates of endometrial, ovarian, and colorectal cancer.^30^ Oral contraceptives are well-tolerated, serious side effects are rare, and compliance with use is high.^31^

Venous thromboembolism is a rare complication of OCs.^31^ The first report of increased risk of VTE associated with use of OCs was in 1961^32^ and since then many different studies have confirmed an increase of two to six times in the risk of VTE. The thromboembolic risk of OCs depends on the estrogen dosage and the type of progestogen combined with it.^33^^,^^34^ Oral contraceptives increase the risk of VTE from a baseline rate of 5/10,000 woman-years among non users to 9 to 10/10,000 woman-years among users.^34^ To keep this risk in perspective, it is important to remember that the risk of VTE is 29/10,000 during pregnancy and 300-400/10,000 in puerperium.^35^ These impressive figures confirm that, in many clinical situations, health professionals advising women not to take OCs verges on the irresponsible.

Older OCs, with high estrogen levels (> 50 μg of ethinylestradiol) are linked with a greater risk of VTE than modern OCs (< 50 μg of ethinylestradiol). Notwithstanding, no reduction of risk was confirmed with pills containing 20 μg of ethinylestradiol compared with pills containing 30μg of ethinylestradiol.^34^ Although OCs with less than 35 μg ethinylestradiol provoked fewer side effects related to estrogen, such as nausea and increased breast sensitivity, during the first months of use, a reduction in risk of VTE was not demonstrated.^36^

The risk of VTE among women on OCs is attributed to changes in hemostasis.^37^ Estrogen increases serum concentration of coagulation factors (prothrombin, factors VII, VIII, and X, and fibrinogen) and reduces concentration of anticoagulant factors (protein S and antithrombin).^38^ These changes can have a clinical impact in women with hereditary thrombophilias.^39^ The risk of VTE associated with OCs increases with the estrogen dose, with increased body weight and age, and with reintroduction or change of OC after a withdrawal exceeding 4 weeks.^34^ The type of progestogens also influences the risk of VTE, and second-generation progestogens (levonorgestrel [LNG] and norethisterone) are safer than third and fourth-generation ones.^40^ The new generations of progestogens are partly responsible for the non-contraceptive benefits of OCs.^31^ Oral contraceptives containing third-generation progestogens have fewer androgenic effects and possibly lower metabolic and cardiovascular risk, but possibly with an increased risk of VTE.^31^

Among users of OCs, those with hereditary thrombophilia, smokers, and the obese are at higher risk of VTE. Recognizing this increased risk, the World Health Organization (WHO) advises against use of OCs by women with these factors. However, because of the low prevalence of hereditary thrombophilias and the high cost of screening for them, routine testing for thrombophilias is not recommended by the WHO.^41^

Presence of a family history of VTE is a strong and common risk factor for OC-linked VTE, even in the absence of hereditary thrombophilias.^42^ The risk of VTE for a woman who takes OC and has a family history of VTE is 15.3 times higher than for a woman who do not take OCs and does not have a family history of VTE.^43^

There is no consensus on the best OC to prescribe. There is, however, an understanding that second-generation progestogens should be the first choice for the majority of women.^44^^,^^45^

Non-oral contraceptives, including patches and vaginal rings, are also associated with increased risk of VTE, raising the risk of VTE by 7.9 and 6.5 times, respectively.^45^ Several different studies report that the risk of VTE is higher during the first year of use, probably because of undiagnosed thrombophilias.^46^ Coagulation does not exhibit significant changes with progesterone-only OCs, implants containing LNG, or medroxyprogesterone injection in depot form.^47^ Normal or increased sensitivity to active C protein was reported 3 months after insertion of the levonorgestrel intrauterine system (LNG-IUS), indicating that this contraceptive does not have pro-thrombotic effects.^48^

### Venous thromboembolism and assisted fertilization

Infertility affects more than 10% of couples worldwide.^49^^,^^50^ In vitro fertilization (IVF) is a process in which ovarian cells are fertilized by spermatozoa outside of the body and is the most widely-used technique for human reproduction in infertile couples.^51^^,^^52^ Venous thromboembolism is a rare complication in women who undergo IVF and occurs in 0.1 to 2.4% in each fertilization cycle.^53^^-^57 The risk of VTE is double during prenatal period after IVF (odds ratio [OR] 2.18; 95% confidence interval [95%CI] 1.63-2.92), when compared with the baseline risk of other pregnant women. This is because of a five-to-ten-times increase in the risk of VTE during the first trimester of gestations after IVF, partly because of the very high risk of VTE in ovarian hyperstimulation syndrome (OHS), which is a iatrogenic and potentially fatal complication that occurs in 33% of all cycles generated by IVF.^58^ The incidence of VTE after IVF is 0.1% in cycles without OHS and from 0.8% to 2.4% of those in which OHS occurs.^53^^-^57 Women who have OHS are at 100 times greater risk of VTE^59^ and, in severe OHS, thromboprophylaxis with LMWH reduces 26 cases of VTE for every 1,000 women treated (number necessary to treat [NNT] is 39, based on a baseline VTE risk of 4%), without significant increase in bleeding.^24^ Venous thromboembolism occurs with greatest frequency between the 40th and 42nd days after transfer of the embryo. In vitro fertilization also increases the risk of arterial thrombosis, which occurs earlier, on average on the 10th day after transfer of the embryo.^58^^,^^60^^,^^61^

Venous thromboembolism associated with IVF has a propensity to sites in the upper extremities and the cervical region, rather than in the left lower limb, as is more common in spontaneous pregnancies.^60^^-^67

In addition to OHS, other obstetric complications are related to a greater risk of VTE in patients who undergo IVF: multiple gestations, prematurity, fetal death, preeclampsia, and prenatal and postpartum hemorrhage.^58^^-^65^,^^68^

### Venous thromboembolism and hormone replacement therapy

Venous thromboembolism is an uncommon event before menopause, but incidence increases considerably after this period, at around one event per 1,000 woman-years at around 50 years of age, and mortality is 10% in these cases.^69^^-^73

Risk factors for VTE include genetic predisposition (hereditary thrombophilias), constitutional factors (age, overweight, and obesity), comorbidities (cancer, heart failure, active systemic lupus erythematosus, antiphospholipid antibody syndrome, inflammatory polyarthropathy, inflammatory intestinal disease, nephrosis, diabetes mellitus type I with nephropathy, sickle-cell anemia, and intravenous drug use), use of tamoxifen, immobility, and surgery.

Although recent data show that the risks could outweigh the benefits for women who take hormone replacement therapy (HRT), many are still prescribed estrogens to minimize symptoms of the menopause, which can be an additional risk factor for VTE. Women who have a uterus are also given progestogens to neutralize the risk of endometrial cancer.^69^^,^^74^^,^^75^

A great variety of HRT can be used for women going through the menopause and the preparations can differ in terms of their adverse effects. There is evidence that the risk of VTE among users is dependent on the route of estrogen administration. In fact, the transdermal route is not associated with an increased risk of VTE among postmenopausal women.^71^^,^^75^^-^86 Additionally, the type of concomitant progestogens was recently identified as an additional VTE risk factor in women who take HRT.^75^^,^^87^

Observational studies, systematic reviews, and metanalyses consistently report a two to three times greater risk of VTE among postmenopausal women on HRT.^71^^,^^75^^,^^88^^-^93 Women who have used HRT in the past have a similar VTE risk to those who have never used the treatment and, among those on HRT, risk is greatest during the first year of treatment. However, there are no consistent data on risk of VTE according to the method of HRT, including type and dose of estrogens, route of administration, and the potential role of progestogens.^69^^,^^75^

In order to prevent VTE in women who request HRT, it is important to identify susceptible subsets. Hereditary thrombophilias are well-established risk factors for VTE, increasing the risk by three times in postmenopausal women (OR 3.3; 95%CI 2.6-4.1).^88^ The combination of these mutations with estrogen taken orally increases the risk of VTE (OR 8.0; 95%CI 5.4-11.9) compared to the risk among women without these mutations and not taking estrogen.^75^ However, there was no difference in risk of VTE among women using the transdermal route when compared with women who did not use HRT.^88^

Women with a personal and family history of VTE are considered high risk and, therefore, are not candidates for HRT with oral route estrogen. Oral route estrogen provokes procoagulatory changes, such as increased resistance to active C protein, by reducing serum concentration of protein S, probably because of passage of estrogen through the liver and reduction of fibrinolytic activity.^75^^,^^94^

These changes are not observed with the transdermal route. The impact of progestogens in postmenopausal women on HRT has been studied little.^95^^-^97

The Menopause, Estrogen and Venous Events Study (MEVE) investigated the association between transdermal estrogen and risk of VTE recurrence in 1,023 postmenopausal women with prior history of VTE.^98^ The results suggest that transdermal estrogen is safe with regard to VTE recurrence and confirmed that oral estrogen increased the risk of recurrence (relative risk [RR] 6.4; 95%CI 1.59-27.3).

Hormone replacement therapy is the most effective treatment for climacteric symptoms associated with falling estrogen levels after menopause.^74^^,^^75^ After evaluation of the risks and benefits, HRT should be prescribed with the lowest estrogen dose and shortest duration possible. Current data do not support its use for primary or secondary prevention of cardiovascular diseases or dementia.^71^^,^^75^^,^^98^ Since PE is the greatest cause of death attributed to HRT among postmenopausal women aged 50-59 years, the reduced risk of VTE linked with HRT using transdermal estrogen alone or combined with micronized progestogen indicates that this is the most recommended strategy for improving the risk-benefit relationship of HRT.^71^ This strategy is confirmed in the North American Menopause Society and the European Menopause Society guidelines.^75^^,^^99^^,^^100^

## CONCLUSIONS

Venous thromboembolism is a current challenge in obstetric practice, particularly after the reductions in hemorrhagic complications and infectious during pregnancy and puerperium observed in more developed settings. Preventative interventions of a mechanical and pharmacological nature reduce occurrence of VTE and its short and long-term complications. The implementation of several prophylaxis guidelines and protocols reflect concern with the quality of care provided to pregnant women. Irrespective, adequate attention to contraception and HRT also demand maturity and knowledge. Simply prohibiting use of OCs and HRT without carefully assessing risk factors and family and personal history, does not decisively combat occurrence of VTE and unnecessarily exposes woman to a risk of reduced quality of life.
