# Supplementary material for: Venous thromboembolism in women: new challenges for an old disease
Source: J Vasc Bras. 2020 Jul 6;19:e20190148. doi: 10.1590/1677-5449.190148 (PMC8202191; doi:10.1590/1677-5449.190148)
Supplement: Translation

# Translation: Tromboembolismo venoso na mulher: novos desafios para uma velha doença

**Authors:** André Luiz Malavasi Longo de Oliveira, Adilson Ferraz Paschôa, Marcos Arêas Marques

**Affiliations:** 1 Centro de Referência da Saúde da Mulher do Estado de São Paulo, Obstetrícia e Ginecologia, São Paulo, SP, Brasil.; 2 Hospital da Beneficência Portuguesa de São Paulo, Cirurgia Vascular, São Paulo, SP, Brasil.; 3 Universidade do Estado do Rio de Janeiro – UERJ, Hospital Universitário Pedro Ernesto, Unidade Docente Assistencial de Angiologia, Rio de Janeiro, RJ, Brasil.; 4 Universidade Federal do Estado do Rio de Janeiro – UNIRIO, Hospital Universitário Gafrée e Guinle, Serviço de Cirurgia Vascular, Rio de Janeiro, RJ, Brasil.

**Keywords:** trombose, anticoncepcionais, gravidez, terapia de reposição hormonal, mulheres, embolia pulmonar

## Abstract

Nos países que controlaram as causas clássicas de óbito materno, como eclâmpsia e hemorragia, o tromboembolismo venoso (TEV) passou a ser a principal preocupação. A prevenção do TEV na gestação e no puerpério, por meio de diretrizes e da instituição de farmacoprofilaxia, é ainda a melhor estratégia para reduzir essa complicação. Os contraceptivos hormonais e a terapia de reposição hormonal também aumentam o risco de TEV; porém, as mulheres não podem ser privadas dos benefícios dessas terapias, que as tornam mais livres na idade fértil e menos sintomáticas na menopausa. Tanto o uso indiscriminado quanto a proibição imotivada são inadequados. A escolha dos métodos contraceptivos e de reposição deve ser feita por criteriosa seleção, avaliando as contraindicações, os critérios de elegibilidade e a autonomia das pacientes. O presente artigo apresenta uma revisão não sistemática da literatura recente visando a avaliar e resumir a associação entre TEV e situações clínicas peculiares ao sexo feminino.

## TROMBOEMBOLISMO VENOSO NA GESTAÇÃO

A obstetrícia moderna enfrenta o dilema da dualidade sangramento-tromboembolismo. Se, no passado não muito remoto, o grande temor dos que assistiam à parturiente era a hemorragia acidental, hoje esse receio é acrescido de mais um complicador: o tromboembolismo venoso (TEV)^1^^,^^2^.

A gestante apresenta os três componentes etiopatogênicos da tríade de Virchow: a) estase venosa, devido à compressão das veias cava e ilíaca comum esquerda pelo útero gravídico e à diminuição do tônus venoso por causa da ação miorrelaxante da progesterona; b) hipercoagulabilidade, secundária à indução da síntese hepática dos fatores VII, VIII e X de coagulação pelo estriol placentário, aumento do fibrinogênio e do inibidor do ativador do plasminogênio tipo I e II, e diminuição da síntese de proteína S; c) lesão endotelial, que ocorre na nidação, na remodelação endovascular das artérias uteroespiraladas, e na dequitação^3^.

Durante a gestação, o risco de TEV aumenta de cinco a dez vezes, podendo chegar a 35 vezes no puerpério, quando comparado ao de mulheres não gestantes de mesma idade^4^. Após esse período, a frequência diminui rapidamente; porém, um risco residual permanece por até 12 semanas após o parto^5^.

A trombose venosa profunda (TVP) de membros inferiores é responsável por 75 a 80% dos episódios de TEV na gestação. Aproximadamente dois terços das TVPs ocorrem no período antenatal e distribuem-se igualmente nos três trimestres. Entretanto, de 43 a 60% dos episódios de embolia pulmonar (EP) ocorrem nas primeiras 6 semanas do puerpério^6^. Nas gestantes, as TVPs predominam ainda mais no membro inferior esquerdo (90% *versus* 55%) e no segmento íleo-femoral (72% *versus* 9%), quando comparadas às não gestantes. Esse fato pode ser explicado pela acentuação da compressão da veia ilíaca comum esquerda pela artéria ilíaca comum direita contra a quinta vértebra lombar, causada pelo útero gravídico^3^.

A prevalência do TEV é de 0,5 a 2,2 casos para cada 1.000 partos, dependendo da população estudada^7^, e a incidência absoluta de TEV na gestação e no puerpério foi de 107 por 100.000 mulheres-ano no Reino Unido (RU)^8^ e de 175 por 100.000 mulheres-ano na Dinamarca e no Canadá^9^. No Brasil, não há dados oficiais sobre a mortalidade materna por TEV^6^.

A EP permanece como a principal causa de morte materna direta no RU; porém, houve uma queda significativa no parto vaginal, de 1,56 por 100.000 partos em 2003-2005 para 0,70 por 100.000 partos em 2006-2008, justificada pela aplicação da primeira versão (2004), das diretrizes do *Royal College of Obstetricians and Gynaecologists* (RCOG) para redução do risco de TEV na gestação e no puerpério^10^. A prevenção do TEV na gestação, por meio da aplicação de diretrizes, e a consequente instituição de profilaxia mecânica e/ou farmacológica, é a melhor estratégia para reduzir essa intercorrência^6^.

## FATORES DE RISCO DE TEV DURANTE A GESTAÇÃO

### Sobrepeso e obesidade

A obesidade [índice de massa corporal (IMC) > 30 kg/m^2^] é um importante fator de risco para TEV na gestação, e esse risco cresce à medida que o IMC aumenta^11^, chegando a ser 14,9 vezes maior quando comparado ao de mulheres não obesas^12^. Entre as gestantes que morreram de EP no RU entre 2003 e 2008, 60% eram obesas^10^. O sobrepeso materno (IMC entre 25 e 29,9 kg/m^2^), apesar de comum, também é considerado um fator de risco menos importante para TEV relacionado à gestação^12^.

### Idade

Dados extraídos de estudos do tipo caso-controle sugerem aumento de risco de duas vezes para mulheres acima de 35 anos^13^. Em estudo conduzido no RU, em que se utilizou uma coorte ampla de mulheres fora da gestação, aquelas com idade entre 35 e 44 anos apresentaram risco 50% maior de TEV quando comparadas com aquelas entre 25 e 34 anos. A ocorrência de TEV não aumentou com a idade no período anteparto; contudo, mulheres em fase puerperal entre 35 e 44 anos apresentaram risco 70% superior quando comparadas àquelas entre 25 e 34 anos (um aumento de risco absoluto de 1,6 por 1.000 pessoas-anos)^8^. De modo geral, considera-se a idade de 35 anos ou mais como fator de risco antenatal e puerperal^14^.

### Trombofilias

De fato, existe a associação entre trombofilias hereditárias e TEV; porém, a pesquisa sistemática de trombofilias não deve ser realizada, e sim restrita à pacientes com episódio pregresso e história familiar de trombofilia com maior potencial de complicação, como deficiência de antitrombina e homozigose do fator V Leiden e mutação 20210 da protrombina, que podem exigir a profilaxia do TEV. Esta, a depender das circunstâncias, poderá ser indicada desde o início da gravidez até o puerpério, a partir da 28ª semana, ou apenas durante o puerpério. É importante ressaltar que a história clínica é mais relevante para essa decisão do que o resultado da pesquisa laboratorial. Quanto à associação entre trombofilias de causas genéticas e trombose uteroplacentária que ocasione perda fetal, pré-eclâmpsia, restrição de crescimento fetal ou descolamento prematuro de placenta, é um assunto ainda controverso^15^^-^17.

### Viagens de longa distância e imobilidade

Os dados relacionados à imobilidade e a viagens de longa distância em gestantes são limitados, sendo necessária a extrapolação de estudos de populações não gestantes^18^. As diretrizes relativas a cuidados antenatais do Instituto Nacional de Excelência em Saúde e Cuidados (*National Institute of Health and Care Excellence*, NICE)^19^ e as recomendações do RCOG sobre viagens aéreas durante a gestação estabelecem que voos com duração superior a 4 horas aumentam o risco de TEV. Um estudo de caso-controle norueguês apontou aumento do risco de TEV em gestantes com IMC > 25 kg/m^2^ e imobilização anteparto (definida como restrição ao leito por tempo igual ou superior a uma semana antes do parto ou antes do diagnóstico de TEV), mostrando efeito multiplicador sobre o risco de TEV anteparto e pós-parto^11^.

A admissão hospitalar durante a gravidez é associada a aumento de 18 vezes no risco de TEV em comparação ao risco basal fora do hospital, e o risco permanece elevado após o parto, nos 28 dias seguintes. Na internação hospitalar, o risco é maior no terceiro trimestre de gravidez e em mulheres acima de 35 anos^20^.

### Outros

Algumas comorbidades têm sido associadas ao aumento do risco de TEV durante a gestação, entre elas estão: doença intestinal inflamatória, infecção do trato urinário, lúpus eritematoso sistêmico, cardiopatias, hipertensão arterial sistêmica induzida pela gestação ou pré-eclampsia, e cirurgia antenatal não obstétrica^21^. Na análise de dados de 1.475.301 altas hospitalares em maternidades escocesas, Kane et al. encontraram fatores de risco associados ao TEV que incluíam três ou mais gestações anteriores, hemorragia obstétrica e pré-eclampsia^21^. A hiperêmese aumenta o risco de TEV ante e pós-natal em 4,4 vezes.

Essas informações têm importantes implicações, já que muitos eventos tromboembólicos são fatais e ocorrem no primeiro trimestre, frequentemente antes da primeira consulta pré-natal, quando poderia ser instituída a profilaxia antenatal^22^.

### Fatores relacionados ao tratamento anticoagulante durante a gestação e o puerpério

O uso da varfarina na gestação pode induzir o descolamento prematuro da placenta, a embriopatia, anormalidades do sistema nervoso central (SNC) e o sangramento fetal. A embriopatia varfarínica é caracterizada pela hipoplasia nasal e/ou pela não consolidação das epífises, e está associada à exposição à varfarina entre a sexta e a 12ª semanas de gestação^23^. As anormalidades do SNC associadas com o uso da varfarina incluem displasia da linha média dorsal com agenesia do corpo caloso, atrofia da linha média cerebelar, displasia da linha média ventral com atrofia óptica e amaurose, e hemorragia, podendo ocorrer após a exposição varfarínica, em qualquer fase da gestação. A varfarina é segura na amamentação^24^.

Duas abordagens podem ser adotadas para diminuir o risco do uso de varfarina em mulheres que necessitam de anticoagulação e desejam engravidar. A primeira é manter a varfarina e realizar testes de gravidez frequentes. Tão logo a gravidez seja diagnosticada antes da sexta semana, a heparina de baixo peso molecular (HBPM) deve ser introduzida no lugar da varfarina. A outra abordagem é suspender a varfarina e iniciar a HBPM logo que a decisão de tentar engravidar seja tomada^24^^-^28.

Apesar de trazerem grandes avanços à profilaxia e ao tratamento do TEV em diversas situações clínicas, os anticoagulantes orais diretos existentes (apixabana, dabigatrana, edoxabana e rivaroxabana) são contraindicados na gestação, por cruzar a barreira placentária, e na amamentação, por passar para o leite materno^6^^,^^23^^-^25.

### Cesariana

Embora o risco de TEV associado à cesariana isoladamente seja baixo, a ocorrência de TEV passa a ser significativa quando há relação com outros fatores de risco, e deve-se indicar a instituição de tromboprofilaxia^29^. Na gestante anticoagulada, o parto deve ser programado entre 37 e 40 semanas. A HBPM deve ser suspensa 12 horas antes do parto se administrada em dose profilática, ou 24 horas antes se administrada em doses intermediária ou plena, o que permitirá a realização da raquianestesia ou da peridural com segurança. Gestantes em uso de HBPM devem ser orientadas a não administrar a dose do fármaco caso apresentem contrações ou perda de líquido^23^.

A indicação da via de parto é obstétrica, não havendo contraindicação à maturação artificial do colo nem à indução do trabalho de parto. Sendo a via de parto vaginal ou cesárea, a paciente deve permanecer com uso de meias antitrombo durante o procedimento.

### Estratificação de risco

A estratificação de risco de TEV na gestação baseia-se na avaliação de cada paciente e deve ser realizada em todas as mulheres com intenção de engravidar ou logo que engravidam. Recomenda-se repeti-la ao longo do pré-natal, diante do surgimento de novos fatores de riscos. As preferências e as considerações das gestantes devem ser levadas em conta no momento da escolha da tromboprofilaxia, muito embora, nesse cenário, as opções terapêuticas sejam restritas^26^.

As recomendações sugeridas dependem de variações individuais entre as pacientes e têm o intuito de informar, e não de substituir, o julgamento clínico do médico, que em última análise deve determinar a conduta apropriada para cada caso. As diretrizes mais relevantes na área de diagnóstico, profilaxia e tratamento do TEV na gestação são as das entidades *American College of Obstetricians and Gynaecologists* (ACOG)^30^, *Society of Obstetricians and Gynaecologists of Canada* (SOGC)^31^, RCOG^27^ e *American College of Chest Physicians* (ACCP)^28^^,^^29^.

### Resumo das diretrizes

Como mencionado anteriormente, as diretrizes têm por objetivo ordenar as condutas mais apropriadas em função das publicações disponíveis na literatura. Todavia, situações atípicas são frequentes e exigem um tratamento diferenciado. A seguir serão apresentadas as principais diretrizes.

**ACOG:** compressão pneumática intermitente (CPI) antes da cesariana se a paciente não fizer uso de tromboprofilaxia^30^.

**SOGC:** recomenda-se farmacoprofilaxia no pós-parto diante das seguintes situações: TEV prévio, trombofilia de alto risco (síndrome do anticorpo antifosfolipídeo, deficiência de antitrombina, homozigose do fator V de Leiden ou mutação do gene G20210A da protrombina ou trombofilias combinadas), restrição ao leito antes do parto por 7 ou mais dias, sangramento maior que 1 L no periparto ou no pós-parto, transfusão de hemoderivados, cirurgia pós-parto e infecção no periparto ou no pós-parto^31^.

Deve-se considerar o uso de farmacoprofilaxia na ocorrência de duas ou mais das seguintes situações: IMC ≥ 30 kg/m^2^ na primeira consulta pré-natal, tabagismo > 10 cigarros/dia, pré-eclâmpsia, restrição de crescimento fetal, placenta prévia, cesariana de emergência, sangramento maior que 1 L no periparto ou no pós-parto ou transfusão de hemoderivados, trombofilia de baixo risco (deficiência de proteínas C ou S, heterozigose do fator V de Leiden ou mutação 20210A do gene da protrombina), doença cardíaca materna, lúpus eritematoso sistêmico, anemia falciforme, doença inflamatória intestinal, varizes de membros inferiores, diabetes gestacional, parto prematuro, parto de natimorto, ou três ou mais dos seguintes fatores de risco: idade > 35 anos, paridade ≥ 2, qualquer técnica de reprodução assistida, gestação múltipla, descolamento prematuro de placenta, ruptura prematura de membranas, cesariana eletiva ou câncer materno.

Mulheres com fatores de risco persistentes devem receber farmacoprofilaxia no mínimo por 6 semanas pós-parto, já as com fatores transitórios no anteparto e intraparto devem receber farmacoprofilaxia até a alta hospitalar, ou até 2 semanas após o parto^25^.

**RCOG:** cesariana de emergência, 10 dias após o parto com dose profilática de HBPM; para todas as outras pacientes submetidas a cesariana, considerar 10 dias de HBPM em dose profilática se houver outros fatores de risco^27^.

**ACCP:** na ausência de fatores de risco adicionais, deambulação precoce; no caso de um fator de risco maior ou de dois ou mais menores (um menor se houver cesariana de emergência), sugere-se profilaxia com HBPM após o parto enquanto a paciente permanecer no hospital (se houver contraindicação à anticoagulação, usar profilaxia mecânica com meias antitrombo ou CPI); no caso de risco altíssimo com fatores de risco adicionais que persistem no puerpério, combinar HBPM com meias antitrombo e/ou CPI; as pacientes selecionadas de alto risco com fatores de risco adicionais que persistem no puerpério devem receber até 6 semanas de extensão de farmacoprofilaxia após a alta hospitalar^28^^,^^29^.

### Tromboembolismo venoso e contracepção

No Brasil, uma em cada cinco mulheres utiliza contraceptivo oral (CO)^32^. Os COs oferecem benefícios além da contracepção, como redução do sangramento menstrual, dismenorreia, tratamento da síndrome pré-menstrual, enxaqueca menstrual, acne e hirsutismo. Os benefícios em longo prazo incluem redução na incidência de câncer endometrial, ovariano e colorretal^30^. Os COs são bem tolerados, com raros efeitos colaterais sérios, e apresentam grande adesão ao uso^31^.

O TEV é uma complicação rara dos COs^31^. O primeiro relato de aumento de risco de TEV associado ao uso de COs foi em 1961^32^, e desde então vários estudos têm confirmado um aumento de duas a seis vezes no risco de TEV. O risco tromboembólico de COs depende da dose de estrógeno e do tipo de progestágeno associado^33^^,^^34^. Os COs aumentam o risco de TEV de uma taxa basal de 5/10.000 mulheres-ano em não usuárias para 9 a 10/10.000 mulheres-ano em usuárias^34^. Para não se perder a perspectiva desse risco, é importante recordar que o risco de TEV é de 29/10.000 na gestação e de 300-400/10.000 no puerpério^35^. Esses números expressivos confirmam que, em várias situações clínicas, o desaconselhamento do uso de COs pelos profissionais de saúde beira a irresponsabilidade.

Os COs antigos, com altos níveis de estrógeno (> 50 μg de etinilestradiol), apresentam risco maior de TEV que os COs modernos (< 50 μg de etinilestradiol). Todavia, a redução de risco de pílulas com 20 μg de etinilestradiol comparadas com as pílulas de 30μg de etinilestradiol não foi confirmada^34^. Os COs com menos de 35 μg de etinilestradiol, apesar de terem menos efeitos colaterais relacionados ao estrógeno, como náuseas e aumento de sensibilidade mamária, nos primeiros meses de uso, não demonstraram redução de risco de TEV^36^.

O risco de TEV em mulheres em uso de COs é atribuído às mudanças na hemostasia^37^. O estrogênio aumenta a concentração sérica dos fatores de coagulação (protrombina, fator VII, VIII, X e fibrinogênio) e reduz a dos fatores anticoagulantes (proteína S e antitrombina)^38^. Essas mudanças podem ter um impacto clínico em mulheres portadoras de trombofilias hereditárias^39^. O risco de TEV associado aos COs aumenta com a dose de estrógeno, com aumento do peso corporal e da idade, e com o reinício ou a troca do CO após interrupção superior a 4 semanas^34^. O tipo de progestágeno também influencia no risco de TEV, sendo os progestágenos de segunda geração [levonorgestrel (LNG) e noretisterona] mais seguras que os de terceira e quarta^40^. As novas gerações de progestágeno são, em parte, responsáveis pelos benefícios não contraceptivos dos COs^31^. Os COs contendo progestágeno de terceira geração apresentam menos efeito androgênico e possivelmente menor risco metabólico e cardiovascular, porém com possível risco aumentado para TEV^31^.

Entre as usuárias de COs, as portadoras de trombofilia hereditária, tabagistas e obesas apresentam risco maior de TEV. Reconhecendo esse aumento de risco, a Organização Mundial da Saúde (OMS) desaconselha o uso de COs em mulheres nessas condições. Entretanto, devido à baixa prevalência de trombofilias hereditárias e aos altos custos de rastreamento, a pesquisa rotineira de trombofilias não é recomendada pela OMS^41^.

A presença de história familiar de TEV é um fator de risco forte e comum para TEV associado ao COs, mesmo na ausência de trombofilias hereditárias^42^. O risco de TEV com uso de CO numa mulher que apresenta história familiar de TEV é 15,3 vezes maior que o de uma mulher não usuária de CO e sem história familiar de TEV^43^.

Não há consenso sobre qual o melhor CO a ser prescrito. Todavia, há entendimento de que os progestágenos de segunda geração devem ser a primeira escolha para a maior parte das mulheres^44^^,^^45^.

Contraceptivos não orais são também associados com aumento de risco de TEV, incluindo os adesivos transdérmicos e o anel vaginal, com aumento de 7,9 a 6,5 vezes de risco de TEV, respectivamente^45^. Vários estudos reportam que o risco de TEV é maior no primeiro ano de uso, provavelmente devido a trombofilias não diagnosticadas^46^. A coagulação não apresenta mudanças significativas com COs exclusivos de progesterona, implantes contendo LNG ou injeção de medroxiprogesterona em forma de depósito^47^. A sensibilidade normal ou aumentada à proteína C ativada foi reportada 3 meses após a inserção de sistema intrauterino de levonorgestrel (SIU-LNG), indicando que esse contraceptivo não tem efeito pró-trombótico^48^.

### Tromboembolismo venoso e fertilização assistida

A infertilidade afeta mais de 10% dos casais em todo o mundo^49^^,^^50^. A fertilização *in vitro* (FIV) é um processo em que as células ovarianas são fertilizadas pelo espermatozoide fora do corpo e é a principal técnica utilizada em reprodução humana de casais inférteis^51^^,^^52^. O TEV é uma complicação rara em pacientes submetidas a FIV, e ocorre em 0,1 a 2,4% delas em cada ciclo de fertilização^53^^-^57. O risco de TEV no anteparto pós-FIV dobra [*odds ratio* (OR) 2,18; intervalo de confiança de 95% (IC95%) 1.63-2.92], quando comparado com o risco basal das outras gestantes. Isso se deve ao aumento de cinco a dez vezes no risco de TEV no primeiro trimestre em gestações pós-FIV, em parte pelo risco muito alto de TEV na síndrome de hiperestimulação ovariana (SHO), uma complicação iatrogênica e potencialmente fatal, que ocorre em 33% de todos os ciclos gerados por FIV^58^. A incidência de TEV após FIV ocorre em 0,1% dos ciclos sem SHO, e em 0,8% a 2,4% daqueles com SHO^53^^-^57. Mulheres que apresentam SHO têm 100 vezes mais risco de TEV^59^ e, na SHO grave, a tromboprofilaxia com HBPM reduz 26 casos de TEV para cada 1.000 mulheres tratadas [número necessário para tratar (NNT) de 39 (dado um risco basal de TEV de 4%)], sem aumento significativo de sangramento^24^. O TEV ocorre com maior frequência entre o 40º e 42º dia após a transferência do embrião. A FIV também aumenta o risco de trombose arterial, que ocorre mais precocemente, em média no 10^o^ dia pós-transferência do embrião^58^^,^^60^^,^^61^.

O TEV associado à FIV tem a propensão de localizar-se em extremidades superiores e na região cervical, em vez de no membro inferior esquerdo, mais frequente nas gestações espontâneas^60^^-^67.

Além da SHO, outras intercorrências obstétricas são relacionadas a um maior risco de TEV em pacientes submetidas a FIV: gestação múltipla, prematuridade, óbito fetal, pré-eclâmpsia e hemorragia ante e pós-parto^58^^-^65^,^^68^.

### Tromboembolismo venoso e terapia de reposição hormonal

O TEV é um evento incomum antes da menopausa, mas com aumento de incidência importante após esse período, de cerca de um evento por 1.000 mulheres-ano em torno de 50 anos de idade e mortalidade de 10% desses casos^69^^-^73.

Os fatores de risco para TEV incluem a predisposição genética (trombofilias hereditárias), fatores constitucionais (idade, sobrepeso e obesidade), comorbidades (câncer, insuficiência cardíaca, lúpus eritematoso sistêmico ativo, síndrome do anticorpo antifosfolipídeo, poliartropatia inflamatória, doença intestinal inflamatória, nefrose, diabetes melito tipo I com nefropatia, doença falciforme, uso de drogas intravenosas), uso de tamoxifeno, imobilidade e cirurgia.

Apesar de dados recentes apontarem que os riscos podem exceder os benefícios em mulheres que realizam terapia de reposição hormonal (TRH), muitas ainda recebem prescrição de estrógenos para minimizar os sintomas do climatério, o que pode ser um fator de risco adicional para TEV. As mulheres com útero também recebem progestágenos para neutralizar o risco de câncer de endométrio^69^^,^^74^^,^^75^.

Uma grande variedade de TRH pode ser usada entre as mulheres climatéricas, e essas preparações podem diferir em relação aos seus efeitos adversos. Há evidência de que o risco de TEV entre as usuárias depende da via de administração de estrogênio. De fato, a via transdérmica não está associada ao risco aumentado de TEV entre mulheres na pós-menopausa^71^^,^^75^^-^86. Além disso, o tipo de progestágeno concomitante emergiu recentemente como fator de risco adicional de TEV em mulheres que usam TRH^75^^,^^87^.

Estudos observacionais, revisões sistemáticas e metanálises relatam consistentemente um risco de duas a três vezes maior de TEV entre mulheres pós-menopáusicas que utilizam TRH^71^^,^^75^^,^^88^^-^93. Usuárias pregressas de TRH têm um risco TEV similar ao das que nunca usaram essa terapia, e, entre as usuárias de TRH, o risco é maior durante o primeiro ano de tratamento. No entanto, não há dados consistentes sobre o risco de TEV de acordo com a modalidade de TRH, incluindo o tipo e a dose de estrógenos, a via de administração e o papel potencial dos progestágenos^69^^,^^75^.

Com o intuito de prevenir o TEV em mulheres que solicitam TRH, é importante identificar os subgrupos suscetíveis. As trombofilias hereditárias são fatores de risco bem estabelecidos para TEV, aumentando o risco em três vezes em mulheres na pós-menopausa (OR 3,3; IC95% 2,6-4,1)^88^. A combinação dessas mutações com estrógeno por via oral aumenta o risco de TEV (OR 8,0; IC95% 5,4-11,9) comparado ao risco de mulheres sem essas mutações e que não usam estrógeno^75^. Todavia, não houve diferença no risco de TEV nessas mulheres quando usaram a via transdérmica quando comparadas às que não usaram^88^.

Mulheres com história pessoal e familiar de TEV são consideradas de alto risco e, portanto, não são candidatas à TRH com estrogênio via oral. O estrógeno via oral provoca mudanças pró-coagulantes, como o aumento da resistência à proteína C ativada, por redução da concentração sérica de proteína S, provavelmente pela passagem do estrogênio no fígado e pela diminuição da atividade fibrinolítica^75^^,^^94^.

Essas alterações não são observadas pela via transdérmica. Em mulheres na pós-menopausa em TRH, o impacto dos progestágenos tem sido pouco estudado^95^^-^97.

O estudo *Menopause, Estrogen and Venous Events Study* (MEVE) investigou a associação entre estrógeno transdérmico e risco de recorrência de TEV em 1.023 mulheres na pós-menopausa e com história pregressa de TEV^98^. Os resultados sugerem que o estrógeno transdérmico é seguro com relação à recorrência de TEV e confirmam que o estrógeno oral aumenta o risco de recorrência [risco relativo (RR) 6,4; IC95% 1,59-27,3].

A TRH é o mais efetivo tratamento para os sintomas climatéricos associados com a queda estrogênica após a menopausa^74^^,^^75^. Após avaliação dos riscos e benefícios, a TRH deve ser prescrita com a dose mais baixa de estrógeno e pelo menor período possível. Os dados atuais não suportam seu uso para prevenção primária ou secundária de doenças cardiovasculares ou demência^71^^,^^75^^,^^98^. Como a EP representa a principal causa de morte atribuída à TRH nas mulheres pós-menopausa com idade entre 50-59 anos, a redução no risco de TEV na TRH com o estrógeno transdérmico isolado ou associado ao progestágeno micronizado demonstra que essa parece ser a estratégia mais recomendada para melhorar a relação de risco-benefício da TRH^71^. Inclusive, essa estratégia é corroborada pelas diretrizes da Sociedade Norte-Americana de Menopausa e da Sociedade Europeia de Menopausa^75^^,^^99^^,^^100^.

## CONCLUSÃO

O TEV é um desafio atual na prática da obstetrícia, particularmente após a redução das complicações hemorrágicas e infecciosas do período gravídico e puerperal, observada em cenários de maior desenvolvimento. As intervenções preventivas de natureza mecânica e farmacológica reduzem a ocorrência do TEV e de suas complicações a curto e longo prazo. A implantação de várias diretrizes e protocolos de profilaxia refletem a preocupação com a qualidade da atenção à parturiente. Independentemente, a atenção adequada à anticoncepção e à TRH também exige maturidade e conhecimento. Simplesmente proibir a utilização de COs e TRH sem avaliar criteriosamente os fatores de risco e a história familiar e pessoal, além de não coibir decisivamente a ocorrência do TEV, expõe a mulher a riscos desnecessários e à perda de qualidade de vida.
